# Segregation analysis in families with Charcot-Marie-Tooth disease allows reclassification of putative disease causing mutations

**DOI:** 10.1186/1471-2350-15-12

**Published:** 2014-01-21

**Authors:** Rune Østern, Toril Fagerheim, Helene Hjellnes, Bjørn Nygård, Svein Ivar Mellgren, Øivind Nilssen

**Affiliations:** 1 Department of Medical Genetics, University Hospital of North-Norway, NO9038 Tromsø, Norway; 2 Department of Clinical Medicine, Neuromuscular Research Group, University of Tromsø, NO9037 Tromsø, Norway; 3 Department of Neurology, University Hospital of North-Norway, NO9038 Tromsø, Norway

**Keywords:** Charcot-Marie-Tooth, Genetic and inherited disorders, Neuromuscular diseases, Mutation analysis, Family investigations

## Abstract

**Background:**

The identification of disease causing, or putative disease causing, mutations in index patients with Charcot-Marie-Tooth disease (CMT) allows for genetic testing of family members. Relevant variants identified in index patients are of either definite, likely or uncertain pathogenicity. The main objective of this study was to make an evaluation of the family investigations performed as part of the assessment of genetic variants of unknown clinical significance (VUS).

**Methods:**

Between 2004 and 2010 molecular genetic family investigations were requested for 87 family members from 41 families harbouring *PMP22dup* or genetic variants in *GJB1*, *MPZ*, *MFN2* and *NEFL*. Relatives were tested for the family mutation and data from the requisitions were evaluated by means of statistical tools.

**Results:**

The results within each indication category are presented and discussed in detail. Twenty-two relatives (9 affected) from eight families were included in the segregation analyses, which invoked reclassification of three *MFN2* mutations, two of which were *de novo* substitutions (c.2146_2148dup, c.692C > T). One *MFN2* substitution was downgraded due to non-segregation (c.1709 A > G), and a *MPZ* substitution (c.103 G > A) upgraded due to segregation with the phenotype in the family.

**Conclusions:**

The results allow for the evaluation of the patient phenotypes ascertained in families, as opposed to the phenotypic descriptions of index patients. They indicate that *de novo MFN2* mutations are regularly found in patients with a classical CMT2 phenotype. They also demonstrate the importance of a precise clinical and neurophysiologic diagnosis of affected family members. This particularly applies for the examination of variants of uncertain clinical significance. Finally, the fact that 14,6% of affected relatives tested for (probable or certain) pathogenic mutations were mutation negative, demonstrates that clinical evaluation alone is not always sufficient in order to determine their diagnosis. We believe that the results will aid in the estimation and planning of resources required for the various aspects of family evaluations in CMT.

## Background

Charcot Marie Tooth disease (CMT) is an inherited peripheral neuropathy and the prevalence of the phenotype has been determined to be 1:2500 in Western Norway and 1 per 1214 in South-eastern Norway (Akershus County) [1,2]. Symptoms of the classical phenotype encompass distal limb weakness and muscular atrophy, tendon areflexia, and sensory loss, most noticeable in the legs. Foot deformities like *pes cavus* and hammertoes are often found [3]. The first symptoms usually occur in the first or second decade and the physical impairments are mostly mild, and with slow progression [4]. The nerve conduction velocity in the median motor nerve (MNCV) is measured to differentiate between the autosomal dominant demyelinating type 1 and the autosomal dominant axonal type 2 (CMT1 < 38 m/s < CMT2). Intermediate forms of CMT are characterized by relatives with MNCVs in both ranges due to both demyelinating and axonal pathology [5]. Autosomal recessive forms (CMT4), and X-linked forms (CMTX) are designated by the inheritance pattern, independent of NCS results. Within each CMT category subclasses are determined by the disease associated gene or locus, and more than 40 CMT associated genes and loci have been identified [6].

More than 90% of the mutations detected in a diagnostic setting are constituted by a *PMP22dup,* or a sequence variant in the *MPZ* or the *MFN2* gene, causing autosomal dominant CMT, or a mutation in the *GJB1* gene, causing X-linked CMT [7-9]. Patients with a duplication of the *PMP22* region develop a classical CMT phenotype in most cases, but the phenotype is also characterized by inequality in severity, even between close relatives [10]. The median MNCV is always < 38 m/s [10-13]. Patients with *MPZ* mutations may have a grave demyelinating phenotype, Dejerine-Sottas syndrome/CMT3 (DSS, MIM 145900) or congenital hypomyelinating neuropathy (MIM 605253) with very slow MNCVs or they may have a milder CMT1 or CMT2 phenotype with MNCVs in the intermediate or axonal range [14]. *MFN2* mutations may be coupled with the classical CMT2 phenotype, but individuals who experience an early disease onset (<10 years) also tend to have severe symptoms, sometimes also optic atrophy [15,16]. Males with a *GJB1* mutation typically have a more severe phenotype than females, and mostly demonstrate MNCVs in the demyelinating or intermediate range, whereas females commonly are asymptomatic or mildly affected, with MNCVs in the intermediate or axonal range [17].

Relatives of index patients with a documented sequence variant may request genetic counselling and testing. The requests for testing of family members fall within four categories: diagnostic, prenatal, and presymptomatic testing of family members for certain or likely pathogenic variants, and finally, carrier testing/segregation analysis of relatives for the investigation of genetic variants of unknown clinical significance. This study is founded upon a previous report on diagnostic testing, the index patients of this study are described in detail there [9]. The main objective of this study was to make a further assessment of the family investigations performed as part of the evaluation of genetic variants of unknown clinical significance (VUS). We included 87 family members of 41 index patients. Here we provide an overview of the indications for testing, the distribution of relatives under each category as well as the test outcome. We believe that these results will aid in estimating and planning the extent of resources required for the various aspects of family evaluations in CMT performed by diagnostic laboratories. Finally, the results presented show that clinical evaluation alone is not always sufficient in order to determine the diagnosis of affected family members.

## Methods

### Patient population

**Figure 1:**
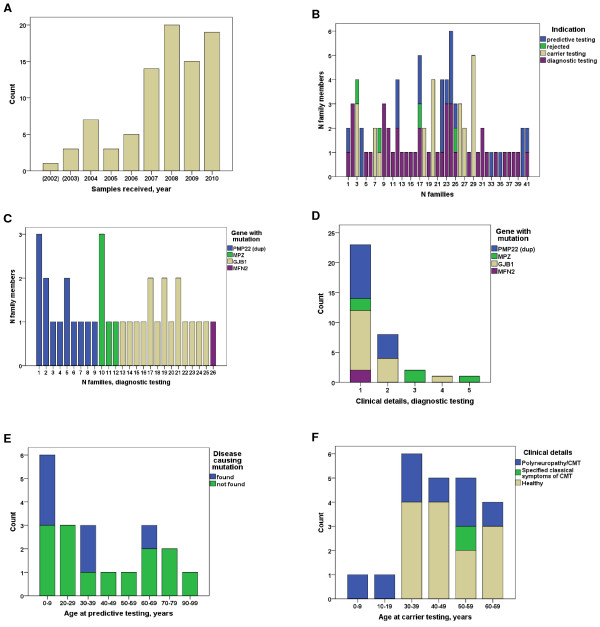
**The quantitative, clinical and genetic characterization of the relatives tested for known family mutations. (A)** The annual number of samples received for family investigations of known genetic variants from 2004–2010. For completeness, 4 individual samples received 2002–2003 were included because their families were otherwise investigated 2004–2010. **(B)** The indications for molecular genetic testing, and the number of relatives within the 41 families. **(C)** The gene variants and number of affected relatives who tested positive within the 26 families. **(D)** Clinical details reported in association with positive testing of affected family members. Group1: Polyneuropathy/CMT without further specifications. Group 2: Detailed description of a classical CMT phenotype. Group 3: As 2, but more severe. Group 4: As in 2, but with additional features that may be seen in association with the investigated genes. Group 5: Description of a phenotype with symptoms that are atypical for the CMT phenotype. **(E)** Age at presymptomatic testing in relation to positive and negative findings. **(F)** Age at testing in family studies of uncertain variants and the proportion of healthy and affected relatives.

Between 2004 and 2010, 435 index patients underwent diagnostic testing for CMT and 72 genetic variants of either definite, likely or uncertain pathogenicity were identified [9]. Subsequent molecular genetic family investigations were requested for 31 families (43%) harbouring *PMP22dup* (6) or mutations in *GJB1* (12), *MPZ* (7), *MFN2* (5) and *NEFL* (1) (Table 1). All mutations were suspected to invoke autosomal dominant or X-linked (*GJB1*) inheritance. For completeness, four individual samples received in 2002 and 2003 were included because their extended families were investigated during the years 2004–2010. In addition, we received samples from 12 relatives of 10 index cases with *PMP22* duplications (9), and a *GJB1* mutation causing p.Arg183Cys, diagnosed in other laboratories. In total, requests for testing of 87 family members from 41 families were received (Figure 1A and B). In 4 cases testing was rejected.

**Table 1:** The 31 index patients diagnosed at our laboratory

***Classification***^***†***^	***Gene***	***N indexes***	***N total***
5	*GJB1*	10	18
	*PMP22*dup	6	
	*MPZ*	2	
4	*GJB1*	2	5
	*MPZ*	2	
	*MFN2*	1	
3	*MPZ*	3	8
	*NEFL*	1	
	*MFN2*	4	

^†^Classification of genetic variants in accordance with the recommendations from the IARC Unclassified Genetic Variants Working Group; 5 = definitely pathogenic, 4 = likely pathogenic, 3 = uncertain, 2 = likely not pathogenic, 1 = definitely not pathogenic [18].

### Genetic analyses

A Genovision M48 (Qiagen) or Biorobot EZ-1 (Qiagen) system was used to extract DNA from peripheral blood cells. Relatives were tested for the specific mutation previously identified in the index patient. Quantitative alterations of the *PMP22* region were assessed by amplification (SALSA MLPA KIT P033-B2 CMT1, MRC Holland, Amsterdam). The Applied Biosystems 3130xl Genetic Analyzer performed the fragment analysis of PCR products. DNA sequencing of the *MPZ*, *GJB1, MFN2* and *NEFL* coding exons including flanking intron sequences were performed as described in Østern et al., 2013 [9].

### Data collection, statistics and endpoint measures

The data sampled from the requisitions and analyzed with the Statistical Package for the Social Sciences (SPSS) version 20.0 included: the sample year and test indication (diagnostic, presymptomatic, carrier, prenatal), clinical details and results on NCS, the age at onset and age at testing, the number of genetically verified affected relatives, the gender of the index patient as well as their relatives, whether or not the mutation was found, and if yes, the name of the gene/mutation and the interpretation of its consequences. The NCS results were classified as demyelinating, axonal, or mixed demyelinating and axonal. The clinical details were divided into: 1: Requests for CMT testing without relevant complementary clinical details, 2: Clinical details supportive of a classical CMT phenotype, 3: As in 2, but significantly more severe, 4: As in 2, but with supplemental traits known to be associated with the genes under investigation (such as tremor, sensorineural hearing impairment), 5: A polyneuropathy phenotype that is atypical for hereditary sensorimotor neuropathy (e.g. HSN-like, HMN-like, prominent upper limb symptoms). (Further details have been published previously [9]). The Alamut software (Interactive Biosoftware, San Diego, CA, USA) and literature studies were used to judge the clinical relevance of the genetic variants. The variants were classified in compliance with the proposal from The IARC Unclassified Genetic Variants Working Group; 5 = definitely pathogenic, 4 = likely pathogenic, 3 = uncertain, 2 = likely not pathogenic, 1 = definitely not pathogenic [18]. Class 3–5 variants were defined as positive findings in this study, whereas class 1–2 variants were defined as negative findings.

With the exception of diagnostic testing, genetic investigations were performed after genetic counselling. Follow up studies of class 3 variants were performed with standard methods, including carrier testing of family members, paternity testing and investigation of control samples [19]. The reclassification of a sequence variant was defined as the adjustment of the interpretation from class 3, to class 2 or 4 after extended investigations. Diagnostic, prenatal, and presymptomatic testing was offered for genetic variants of definite or likely clinical relevance (class 4 and 5).

The study was approved by the Norwegian Data Inspectorate and the Regional Committee for Medical Research Ethics who specified that it was not necessary with informed consent given by subjects/next-of-kin for this study. Procedures were in accordance with the revised Helsinki Declaration of 2008.

## Results

The average number of family members for whom genetic tests were requested was 2.1 per index case (range 1–6). When the laboratory identified a *PMP22* dup- in an index patient, it subsequently received samples of relatives for testing in 23% of the cases. The corresponding percentages for cases harbouring sequence variants in *GJB1*, *MPZ* and *MFN2* were 60%, 64% and 42%, respectively. The indications for molecular genetic testing in the various families and individual family members are illustrated in Figure 1B. In total, 41 positive molecular genetic findings were made (67.2%) in 61 relatives tested for likely or certain pathogenic variants. Furthermore, in three family members and in three index patients from three families, class 3 mutations were upgraded to class 4.

### Diagnostic testing of affected family members for definite or likely pathogenic genetic variants (class 5 and class 4)

Diagnostic testing was performed in 41 affected relatives from 29 families with class 5 or class 4 mutations. Six affected relatives (14.6%), three from families with *PMP22* duplications, one harbouring an *MPZ* mutation and two with *GJB1* mutations, tested negative. NCS results were reported as normal in two and were unspecified in four. For all six, “polyneuropathy” or “CMT” was the only clinical information reported. Age at onset was specified in three cases (5–10, 30–40, 40–50 years), and age at testing was 8, 15, 36, 38, 46 and 54 years respectively.

Thirty-five affected relatives, from 26 families, tested positive for class 4/5 mutations identified in the corresponding index patent (Figure 1C). Average age at testing was 27.6 years (range 2 months – 78 years). NCS results were reported only in 28.6% of the affected relatives.

Only scarce clinical information was given for 65.7% of the affected relatives; such as “polyneuropathy” or “CMT” (clinical group 1) (Figure 1D). The remaining majority (22.9%) had specified a classical CMT phenotype (clinical group 2). In a family with an *MPZ* mutation two patients were classified as severe (clinical group 3) and one as atypical (clinical group 5). This family harboured a c.368C > A (p.Gly123Val) substitution in the *MPZ* gene. The index mother and two of her sons had Dejerine-Sottas syndrome, and a daughter was admitted to the intensive care unit with hypotonia and respiratory difficulties at the age of two months. Another exception was a 42 year old female with CMT and a c.490C > T (p.Arg164Trp) mutation in the *GJB1* gene who had tremor from early childhood, particularly of the head, muscular cramps and fasciculation’s (clinical group 4). The *MFN2* sequence variant, c.653 T > C (p.Leu218Pro), was identified in a mother and her 16 year old son with CMT2 [9]. They had an extensive family history of polyneuropathy; however, other family members were not available for testing. The p.Leu218Pro residue is situated in the important GTPase domain of the protein and is conserved down to *c.elegans* and was interpreted as a class 4 variant based on *in silico* analyses.

### Presymptomatic testing of healthy family members for definite or likely pathogenic genetic variants (class 5 and class 4)

Presymptomatic testing was performed in 20 healthy relatives from 12 families with class 5 or class 4 mutations. Average age at testing was 37.0 years (range 2 – 92 years, Figure 1E). In six minors, aged 15 or younger, for whom NCS data were not available, one *PMP22dup* and two *GJB1* mutations were detected. In 14 relatives, aged 16 years or older, two with normal NCS results and 12 for whom NCS results were lacking, one *PMP22dup* and two *GJB1* mutations were detected.

### Relatives included in the control group for the assessment of genetic variants of unknown clinical significance documented in the index patient (class 3)

In order to evaluate genetic variants of unknown significance carrier testing and segregation analyses were carried out in 22 relatives from eight families. Of nine clinically affected relatives NCS results were available for three (Figure 1F). Average age at testing was 44.7 years (range 3 – 66 years). The patients were older than 30 years with the exception of two cases, a three year old affected girl was tested for a c.410G > A (p.Gly137Asp) mutation in the *MPZ* gene, and a 12 year old affected girl was tested for a c.2146_2148 dup (p.Ala716dup) mutation in the *MFN2* gene. Eight class 3 sequence variants, and their secondary classification after family studies, are listed in Table 2. After segregation studies the classification of mutation pathogenicity was upgraded from class 3 to class 4 for two independent *MFN2* mutations as they showed to have occurred *de novo* in one isolated case of CMT2 and in a patient with an affected carrier child. One *MFN2* mutation was downgraded from class 3 to class 2 since it was also detected in two healthy family members aged 36 and 65 years. Finally, one *MPZ* mutation could be upgraded from class 3 to class 4 after the detection of the same mutation in an affected person remotely related to an index patient.

**Table 2:** Class 3 sequence variants not reported in the HGMDp database or other sources at the time of identification, and secondary classification after family studies

**Gene**	**Family**	**Mutation**		**N tested total**	**N affected tested**^**†**^		**N healthy tested**^**†**^		**Primary classifi-cation**^**$**^	**Secondary classifi-cation**^**£**^
		**cDNA**	**Protein**		**Pos**	**Neg**	**Pos**	**Neg**		
*MFN2*	1^*^	c.250 A > G	p.Lys84Glu	2	0	0	0	2^‡^	3	3
	2	c.1709 A > G	p.Asn570Ser	4	1	0	2	1	3	2^¶^
	3^*^	c.2146_2148 dup	p.Ala716dup	3	1	0	0	2^‡^	3	4
	4^*^	c.692C > T	p.Ser231Phe	2	0	0	0	2^‡^	3	4
*MPZ*	1^*^	c.410 G > A	p.Gly137Asp	3	3	0	0	0	3	3
	2	c.103 G > A	p.Asp35Asn	3	2^€^	0	0	1	3	4
	3^*^	c.368 G > T	p.Gly123Val	5	2	0	0	3	3	3
*NEFL*	1^*^	c.1027_1029del	p.Asp343del	1	1	0	0	0	3	3

^†^In addition to index.

^$^Primary classification of genetic variants in the index patient in accordance with the recommendations from the IARC Unclassified Genetic Variants Working Group; 4 = likely disease causing, 3 = uncertain, 2 = likely not disease causing [18].

^£^Secondary classification of genetic variants after extended family investigations.

^*^Sequence variants reported in a previous work [9].

^‡^Parents negative, *de novo* mutation in the first following generation. *MFN2* family 1; ongoing investigation, *MFN2* family 3 and 4; mutation not found in 200 control chromosomes. Paternity was genetically verified.

^¶^This variant was later reported with a possible association to dHMN in a single Norwegian patient [24].

^€^One affected carrier remotely related to an index patient identified in 2012; not included in the total material.

Altogether three cases of *de novo MFN2* mutations were identified [9]. The first index patient, harbouring a *de novo* c.2146_2148dup (p.Ala716dup) mutation, had normal motor development up to the age of eight years. The patient then had a slowly progressing polyneuropathy with development of *pes cavus* and hammertoes. At the age of 35 the patient had bilateral drop foot, and was still fully employed. The child of the patient was investigated clinically at the age of three due to parental concerns, but was without muscular weakness at the time of assessment. However, NCS and EMG performed six months later were compatible with axonal polyneuropathy. The second patient with a *de novo* c.692C > T (p.Ser231Phe) substitution in the *MFN2* gene had a clinical development similar to that of the index patient with the c.2146_2148dup mutation. The third *de novo* mutation, a c.250A > G (p.Lys84Glu) substitution in the *MFN2* gene, was identified in a 12 year old child with severe mixed (axonal and demyelinating) polyneuropathy, scoliosis, contractures, respiratory difficulties and encephalopathy. None of the patients had optic atrophy.

### Prenatal testing

Nine female carriers at fertile age (21–42 years) were carriers of class 5 variants. Prenatal testing was requested in one case.

## Discussion

In our experience clinicians and diagnostic laboratories have a low threshold for requesting/carrying out diagnostic testing of relatives with polyneuropathy. Accordingly, mutations were not identified in all affected family members (85.4%). This may in part relate to the fact that NCS results were available only for a minority (28.6%). One might anticipate a wider phenotypic spectrum among affected relatives as compared to index patients. However, this was not the case in the material presented here. Additional tremor and fasciculation’s, as reported in a patient with a *GJB1* mutation, or hypotonia and possibly a severe phenotype, as reported in an infant from a DSS family with a *MPZ* mutation, is within the known range of phenotypes. For the remaining affected relatives symptoms of classical CMT were reported. Mild symptoms were reported in two out of three cases with *de novo* mutation in *MFN2*, and age at onset < 10 years of age, whereas the third patient had a phenotype more in accordance with the early onset group, as it is described elsewhere [15,16,20]. Thus, the phenotypes described in this cohort of affected relatives rather show a tendency towards the mild end of the spectrum, particularly in those carrying *MFN2* mutations.

Presymptomatic testing of adult family members gave a detection rate (21%) well below 50%. This might be explained by the high mean age at testing (37.0 years), cases of *de novo* mutations and the inclusion of second degree relatives (3). *De novo* mutations in *GJB1* are assumed to be rare [21]. Minors constituted 30% (6/20) of the presymptomatic group in this material. Five patients had an affected parent, one an affected half sibling. Three of these patients showed a positive genetic test result.

Family investigations and segregation analyses are performed when genetic variants of uncertain clinical relevance (class 3) are revealed and in some cases, interpretations can be modified into class 1/2 or class 4/5, permitting more accurate advice in genetic counselling. Although the families described here are small, they sometimes have the power to weaken the suspicion of pathogenicity. The reclassification of variants identified in nuclear families is mostly achieved in isolated cases through the documentation of a *de novo* mutation in a known CMT associated gene [19]. In four of the eight families with class 3 variants interpretations were adjusted, three of them to class 4, of which two harbored *de novo* mutations in the *MFN2* gene (Table 2). Numerous *de novo MFN2* mutations have been reported in several cohorts with severe symptoms, and some *MFN2* mutations, for example c.280C > T, have also been reported as *de novo* multiple times [15,16,22,23]. Our results therefore add to the data indicating that *de novo MFN2* mutations are regularly found even in patients with a classical CMT2 phenotype [24].

When selecting healthy relatives for segregation analysis older individuals are preferred. The mean age at testing was higher in this group (44.7 years), as compared to the presymptomatic (37.0 years) and diagnostic (28.4 years) groups. An earlier CMT report indicated that many patients, and family members at risk, are uneducated about the possibility of genetic counselling and genetic testing [25]. We do not know to which extent this applies for the cohort presented here, but we do see that significantly more relatives are tested pr. index patient in the group included for segregation analysis (2.7), as compared to the presymptomatic (1.7) and diagnostic (1.4) groups. This probably reflects the more active role of the laboratory in the recruitment of relatives for the investigation of class 3 variants.

Prenatal diagnosis is available in most cases with known class 5 or class 4 variants. In this series only 1/9 women in fertile age requested prenatal testing. Previously we have reported 72 index patients with CMT associated mutations, including 15 females with class 4/5 variants between the age of 20 and 46 years, of whom none asked for prenatal testing at pregnancy [9]. Thus, in our experience, Norwegian female carriers of CMT associated mutations rarely make use of prenatal diagnostics.

## Conclusion

The results presented here clearly demonstrate that the precise diagnosis of affected family members should rely on the combination of NCS and genetic test results, not clinical assessment alone. This particularly applies for the investigation of the clinical significance of class 3 variants. They are coupled with uncertainties that often prevail in spite of meticulous family investigations. The implementation of next generation sequencing technologies in the diagnostic testing of index patients leads to an increase in the already large proportion of class 3 variants. Hence, there is a need for genetic counselling capacity and expertise to be scaled up accordingly to maintain a proportionate relationship between the capacity for diagnostic testing of index patients and the subsequent investigations of their families.

## Competing interests

The authors declare that they have no competing interests.

## Authors’ contributions

RØ has taken part in all stages of the project including design and conceptualization of the study, analysis and interpretation of the data and also in drafting and revising of the manuscript for intellectual content. TF has taken part in analysis and interpretation of the data and also in revising of the manuscript for intellectual content. HH has taken part in analysis and interpretation of the data and in revising of the manuscript for intellectual content. BN has taken part in analysis and interpretation of the data and in revising of the manuscript for intellectual content. SIM has taken part in all stages of the project including design and conceptualization of the study, analysis and interpretation of the data and also in drafting and revising of the manuscript for intellectual content. ØN has taken part in all stages of the project including design and conceptualization of the study, analysis and interpretation of the data and also in drafting and revising of the manuscript for intellectual content. All authors read and approved the final manuscript.

## Pre-publication history

The pre-publication history for this paper can be accessed here:

http://www.biomedcentral.com/1471-2350/15/12/prepub
